# Role of Lactate Dehydrogenase and Lymphocyte Count as Predictors of Poor Perinatal Outcomes in COVID-19-Infected Pregnancies Requiring Hospitalization and Delivery: A Retrospective Cohort Study

**DOI:** 10.7759/cureus.46463

**Published:** 2023-10-04

**Authors:** Sebnem Alanya Tosun, Kivanc Celikkalkan, Alptekin Tosun, Azime Bulut, Enis Ozkaya, Ilknur Senel, Muhammet Bulut, Gokhan Ordu

**Affiliations:** 1 Department of Obstetrics and Gynecology, Giresun University Faculty of Medicine, Giresun, TUR; 2 Department of Pediatrics, Giresun University Faculty of Medicine, Giresun, TUR; 3 Department of Radiology, Giresun University Faculty of Medicine, Giresun, TUR; 4 Department of Anesthesiology, Giresun University Faculty of Medicine, Giresun, TUR; 5 Department of Obstetrics and Gynecology, Zeynep Kamil Maternity and Children Education and Research Hospital, Istanbul, TUR; 6 Department of Infectious Diseases, Giresun University Faculty of Medicine, Giresun, TUR; 7 Department of Obstetrics and Gynecology, Giresun Maternity and Children Education and Research Hospital, Giresun, TUR

**Keywords:** pandemic, neonatal outcome, maternal outcome, lymphocyte, lactate dehydrogenase

## Abstract

Introduction: Physiological, hormonal, or biochemical changes may be related to the increased morbidity of COVID-19 during pregnancy. Our knowledge remains limited about which pregnant women will worsen and develop complications. The aim was to evaluate the maternal, fetal, and neonatal outcomes in hospitalized pregnant women who delivered while infected with acute COVID-19 and to investigate the possible predictors of poor prognosis in a tertiary pandemic center.

Methods: A retrospective cohort study was conducted on pregnant women who required termination or delivery during a COVID-19 infection in a tertiary hospital. Serum markers were analyzed to determine any possible association and the predictive value of these markers to show poor maternal, fetal, and/or neonatal outcomes.

Results: Out of 45 patients, 12 had maternal complications (Group 1) and 33 had no maternal complications (Group 2). The mean lymphocyte at hospital admission was measured as 1,175.83 ± 362.0 and 1,735.30 ± 746.1 in Groups 1 and 2, respectively (p=0.02). The lymphocyte count measured at hospital admission showed significance in predicting poor maternal outcome, with an area under the curve (AUC) of 0.737 (95% CI:0.578 to 0.897) and a cut-off value of 1,110 mcL with 82% sensitivity and 67% specificity. Nineteen of the 45 women had fetal complications. Receiver operating characteristic analysis showed maternal lactate dehydrogenase as a significant predictor for poor fetal outcome with a cut-off value of 213 U/L (AUC:0.719; 95% CI:0.566 to 0.872) with 85% sensitivity and 60% specificity.

Conclusion: The lymphocyte count can be used as a predictor of poor maternal outcome and lactate dehydrogenase demonstrates poor fetal outcome during hospitalization.

## Introduction

Coronavirus disease 19 (COVID-19), caused by an enveloped ribonucleic acid (RNA) virus, is a multisystem disorder that can occur in adults with a wide clinical spectrum from asymptomatic to fatal [1]. Pregnancy is a special period in the reproductive period that shows its own physiologic alterations in the immune and cardiopulmonary systems [2]. Because of the immunosuppressive tendency toward viruses during this period, obstetricians experienced uncertainty when managing pregnant women infected with COVID-19. As far as we know, pregnancy does not make it easier to become infected with COVID-19, but the maternal course may be worse than for healthy women of the same age [3-6]. The Centers for Disease Control and Prevention reported that pregnant women had a higher risk of requiring the intensive care unit (ICU), invasive ventilation, and/or extracorporeal membrane oxygenation, and maternal death rates increased slightly with COVID-19 infection [3]. Conversely, however, in a single study, lower maternal mortality rates were observed due to COVID-19 infection when compared to mortality rates of women of similar ages [7].

Several conditions, such as the weakening of the immune system, the elevation of angiotensin-converting enzyme (ACE)-2 expression, hormonal fluctuations, increased maternal-fetal oxygen needs, and anatomic changes, are considered to be related to the increased morbidity of COVID-19 during pregnancy [8]. Maternal age over 35 years, obesity, metabolic disorders such as diabetes mellitus, and hypertension are relevant risk factors for fatality during pregnancy [9,10]. Although obstetricians may treat serious infections with a fatal course, our knowledge remains limited about which pregnant women will worsen and develop complications. Similarly, the probability of vertical transmission of the virus, which may cause undesired neonatal outcomes, remains uncertain [11]. Unfortunately, the incidence of preterm births and cesarean sections is elevated in COVID-19-infected pregnancies [12].

Essentially, following up on biochemical parameters, using accurate diagnostic guideline protocols, and managing the disease promptly may help prevent perinatal complications with COVID-19 [8]. To the best of our knowledge, there is a lack of predictors to identify poor perinatal outcomes and to focus on defining high-risk patients to help manage their conditions quickly during hospitalization.

Our tertiary hospital is one of the leading pandemic centers in Turkey. The aim of this retrospective study was to evaluate the clinical manifestations, and maternal, fetal, and neonatal outcomes in hospitalized pregnant women who delivered while infected with acute COVID-19 and to investigate the possible predictors of poor prognosis in a tertiary pandemic center.

## Materials and methods

This retrospective cohort study was conducted on pregnant women with COVID-19 who were hospitalized and required termination or delivery at the Giresun University Maternal and Children Education and Research Hospital between March 15, 2020 and February 2021. Our center is a tertiary hospital and is the only center in the region that accepts pregnant women with COVID-19. Inclusion criteria were pregnancies with severe acute respiratory syndrome coronavirus 2 (SARS-CoV-2) positivity diagnosed in a real-time polymerase chain reaction (RT-PCR) assay of nasopharynx and oropharynx swab specimens, pregnancies that required hospitalization, and pregnancies that were terminated or delivered during acute infection. Exclusion criteria were negative RT-PCR cases and pregnancies that did not require hospitalization and/or delivery during infection. This retrospective design study was approved by both the Institutional Ethical Committee of Giresun University (April 3, 2021/15) and the Turkish Ministry of Health (2021-01-10T22_34_17).

A total of 45 patients were evaluated in two groups: pregnancies with maternal complications (Group 1, n = 12) and those without complications (Group 2, n = 33). Maternal complications were classified as mortality, sepsis, the requirement of mechanical ventilation, persistent respiratory distress confirmed by radiologic findings, intracranial nonpyogenic thrombus, and epilepsy after COVID-19 infection. Fetal complications were classified as intrauterine fetal death, prematurity, small for gestational age (SGA) and large for gestational age (LGA). All patients’ sociodemographic and clinical information was recorded as maternal age; gravidity; parity; prepregnancy weight; gestational week and trimester during infection; duration of hospitalization on the ward and ICU; intubation requirement; comorbid diseases; presence of influenza vaccination; maternal symptoms; abnormal vital signs, especially fever; medications used for COVID-19 infection; pregnancy, maternal and, fetal complications; and mode of delivery (vaginal route or cesarean section). Initial laboratory test results, radiologic imaging findings, and blood group were assessed within the first 24 hours following hospitalization. The risk of morbidity and mortality because of sepsis was evaluated using Sequential Organ Failure Assessment (SOFA) scores.

The neonatal outcomes were recorded as gestational week at birth, birth weight, APGAR scores, umbilical cord PH, initial laboratory test results, radiologic imaging findings, and blood group. All biochemical laboratory tests were measured in a single center using chemiluminescence immunoassays with a Roche autoanalyzer.

Some serum markers were analyzed to determine any possible association and the predictive value of these markers, including white blood cells (WBC), lymphocytes, thrombocytes, hemoglobin, aspartate aminotransferase (AST), alanine aminotransferase (ALT), lactate dehydrogenase (LDH), urea, creatine, blood group, C-reactive protein (CRP), D-dimer, vitamin D, vitamin B12, SOFA, procalcitonin, partial pressure of oxygen (PaO_2_), partial pressure of carbon dioxide (PaCO_2_), fibrinogen, ferritin, prothrombin time (PT), activated partial thromboplastin time (aPTT), and international normalized ratio (INR).

Statistical analysis

Statistical analyses were performed using the SPSS version 22 software program (IBM Corp., Armonk, NY). Mean and standard deviation (SD) values were used for normally distributed data. Categorical data are presented as counts and percentages. We used the Mann-Whitney U test to compare continuous variables which were not normally distributed. Several variables were significantly different between the groups, and all these variables underwent multivariate and receiver operating characteristics (ROC) analysis to evaluate the predictive value of the test and to maintain sensitivity and specificity. The most powerful predictors are presented in the Results section. Significance was accepted as P-values < 0.05.

## Results

Baseline characteristics

A total of 45 pregnant women who required termination or delivery during a COVID-19 infection in our tertiary hospital were evaluated. These patients included 22 (48.89%) term deliveries, 14 (31.11%) preterm deliveries, five (11.11%) early preterm (< 32 gestational weeks) deliveries, three (6.67%) terminations of intrauterine fetal death, one (2.22%) abortion, and a total of 41 newborns. Nine (20%) women had comorbidities (diabetes mellitus, hypertension, obesity, and asthma), and only six (13.3%) patients who had no comorbidity had received an influenza vaccination in the past year. Myalgia (n = 31, 68.9%), fever (n = 25, 55.6%), and taste loss (n = 25, 55.6%) were the most common maternal symptoms.

Twelve women had maternal complications (Group 1), and 33 women had no maternal complications (Group 2). The demographic features of the pregnant women in both groups are presented in Table 1. Maternal complications were mortality (n = 1, 2.22%), sepsis (n = 2, 4.44%), requirement for mechanical ventilation (n = 4, 8.89%), persistent respiratory distress (n = 4, 8.89%), and intracranial nonpyogenic thrombus and epilepsy (n = 1, 2.22%). Of the 12 patients with complications, eight (17.8%) required the ICU, and four (8.89%) needed invasive mechanical ventilation. Maternal mortality was observed in one (2.22%) patient during the postpartum period. All 12 patients with maternal complications had suspicious radiologic imaging findings. Fetal complications were intrauterine fetal death (n = 3, 6.67%), SGA (n = 9, 20%), LGA (n = 2, 4.44%), and prematurity (n = 5, 11.11%). Of 41 live births, the cesarean section rate was 80.49%. Only one neonate was positive for COVID-19, delivered at term and by cesarean section. The rate of admission to the neonatal ICU was 80.49%. About one-quarter (24.4%) of the newborns were fed with breast milk from the beginning of the first day, and 66.7% were fed with breast milk after the fifth day.

**Table 1:** Demographic and clinical features of pregnant women who required termination or delivery during COVID-19 infection

Characteristics	With maternal complication (n = 12)	Without maternal complication (n = 33)	p-value
Age (years)	32.25 ± 4.5	30.18 ± 5.4	< 0.05
Gravidity	2.25 ± 1.5	2.15 ± 1.7	< 0.05
Parity	0.92 ± 0.9	1.00 ± 1.3	< 0.05
Prepregnancy Weight (kg)	75.08 ± 14.8	67.12 ± 10.5	< 0.05
Gestational Age During Infection (weeks)	32.80 ± 3.63	34.92 ± 7.0	= 0.02

Predictive performance

There were significant differences in maternal serum markers between Groups 1 and 2 (Table 2). Similarly, there were significant differences in neonatal features between the groups, classified according to the presence or absence of maternal complications (Table 3).

**Table 2:** Maternal outcomes of pregnant women who required termination or delivery during COVID-19 infection WBC = white blood cell; AST = aspartate aminotransferase; ALT = alanine aminotransferase; LDH = lactate dehydrogenase; CRP = C-reactive protein; SOFA = Sequential Organ Failure Assessment score; PaO_2_ = partial pressure of oxygen; PaCO_2_ = partial pressure of carbon dioxide; PT = prothrombin time; aPTT = activated partial thromboplastin time; INR = international normalized ratio.

Parameters	Maternal complication	n	Mean ± SD	P-value
WBC	No	33	8928.13± 3208.8	< 0.05
	Yes	12	8945.00 ± 4884.3	
Lymphocytes	No	33	1735.30 ± 746.1	= 0.02
	Yes	12	1175.83 ± 362.0	
Thrombocytes	No	33	217,454.55 ± 84611.3	< 0.05
	Yes	12	196,250.00 ± 49,715.8	
Hemoglobin	No	33	13.97 ± 13.3	< 0.05
	Yes	12	11.59 ± 1.3	
AST	No	33	18.24 ± 12.2	< 0.05
	Yes	12	23.08 ± 20.8	
ALT	No	33	15.79 ± 11.9	< 0.05
	Yes	12	27.58 ± 35.3	
LDH	No	33	243.36 ± 111.8	< 0.05
	Yes	12	261.17 ± 56.1	
Urea	No	33	15.21 ± 5.6	< 0.05
	Yes	12	13.17 ± 6.6	
Creatinine	No	33	0.53 ± 0.1	< 0.05
	Yes	12	0.50 ± 0.1	
CRP	No	33	18.86 ± 20.8	< 0.05
	Yes	12	52.07 ± 54.4	
D-dimer	No	30	1487.37 ± 1502.0	< 0.05
	Yes	12	2412.92 ± 2285.3	
Vitamin D	No	23	13.57 ± 9.6	< 0.05
	Yes	11	17.01 ± 9.4	
Vitamin B12	No	24	221.88 ± 113.2	< 0.05
	Yes	11	237.09 ± 86.4	
SOFA	No	33	0.00 ± 0.0	< 0.001
	Yes	12	1.33 ± 1.0	
PaO2	No	28	52.00 ± 16.6	< 0.05
	Yes	12	48.3167 ± 17.8	
PaCO2	No	28	40.0286 ± 7.71	< 0.05
	Yes	12	39.5167 ± 9.7	
Procalcitonin	No	28	0.086 ± 0.11	< 0.05
	Yes	11	0.134 ± 0.14	
Fibrinogen	No	27	383.55 ± 140.2	< 0.05
	Yes	10	389.30 ± 201.6	
Ferritin	No	24	63.31 ± 83.6	< 0.05
	Yes	11	82.30 ± 136.7	
PT	No	33	13.40 ± 2.6	< 0.05
	Yes	12	13.98 ± 1.9	
aPTT	No	33	27.27 ± 4.7	< 0.05
	Yes	12	29.13 ± 4.6	
INR	No	33	1.57 ± 3.0	< 0.05
	Yes	12	1.07 ± 0.2	

**Table 3:** Neonatal outcomes of newborns whose mothers are complicated by COVID-19 infection AST = aspartate aminotransferase; ALT = alanine aminotransferase; LDH = lactate dehydrogenase; CRP = C-reactive protein; PT = prothrombin time; aPTT = activated partial thromboplastin time; INR = international normalized ratio.

Neonatal outcomes	Maternal complication	n	Mean ± SD	P-value
Gestational age during labor (weeks)	No	33	35.95 ± 6.8	= 0.006
	Yes	12	33.46 ± 3.8	
Birth weight (g)	No	32	2981.56 ± 861.4	= 0.01
	Yes	12	2282.08 ± 773.1	
APGAR 1	No	30	7.70 ± 0.6	= 0.05
	Yes	11	6.64 ± 1.7	
APGAR 2	No	30	8.27 ± 0.9	< 0.05
	Yes	11	7.82 ± 1.3	
Umbilical cord pH	No	30	7.28 ± 0.8	= 0.02
	Yes	11	7.33 ± 0.1	
Neonatal Lymphocyte	No	29	7252.41 ± 4788.4	< 0.05
	Yes	11	5808.18 ± 3112.7	
Neonatal AST	No	30	52.93 ± 36.6	= 0.03
	Yes	11	31.82 ± 14.9	
Neonatal ALT	No	30	23.80 ± 32.9	< 0.05
	Yes	11	14.18 ± 13.4	
Neonatal LDH	No	30	606.23 ± 262.4	< 0.05
	Yes	11	610.00 ± 215.2	
Urea	No	30	16.17 ± 10.0	< 0.05
	Yes	11	22.55 ± 24.0	
Neonatal Creatinine	No	30	.582 ± 0.21	< 0.05
	Yes	11	.596 ± 0.17	
Neonatal CRP	No	30	4.67 ± 13.4	< 0.05
	Yes	11	.45 ± 0.4	
Neonatal Procalcitonin	No	26	1.34 ± 2.2	= 0.002
	Yes	11	9.56 ± 10.4	
Neonatal Fibrinogen	No	27	246.78 ± 73.4	
	Yes	10	215.40 ± 98.1	< 0.05
Neonatal D-dimer	No	27	1885.67 ± 1894.1	< 0.05
	Yes	10	1540.70 ± 833.9	
Neonatal Thrombocyte	No	30	283,933.33 ± 14,3310.4	< 0.05
	Yes	11	250,909.09 ± 98,189.1	
Neonatal PT	No	30	17.70 ± 3.1	< 0.05
	Yes	11	18.50 ± 3.2	
Neonatal aPTT	No	30	36.30 ± 9.9	< 0.05
	Yes	11	38.80 ± 7.3	
Neonatal INR	No	30	1.43 ± 0.3	< 0.05
	Yes	11	1.50 ± 0.3	

**Figure 1:**
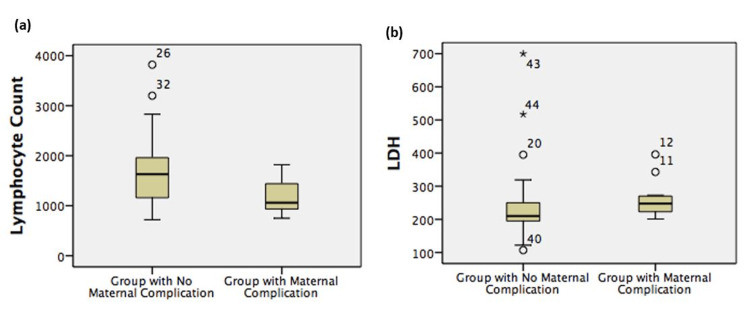
Predictive performance of maternal lymphocyte count and maternal LDH at hospital admission (a) Graphical representation of the mean ± SD values of maternal lymphocyte count; (b) the graphical representation of the mean ± SD values of maternal LDH.

**Figure 2:**
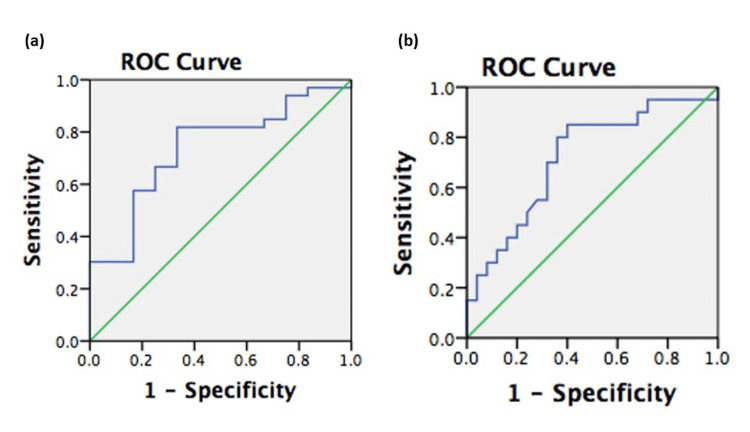
Sensitivity and specificity of predictive values for maternal and fetal complications during COVID-19 infection (a) ROC of lymphocyte count used to distinguish pregnant women with maternal complications during COVID-19 infection; (b) ROC of LDH used to distinguish pregnant women with fetal complications during COVID-19 infection.

The mean maternal lymphocyte count at hospital admission was measured at 1,175.83 ± 362.0 and 1,735.30 ± 746.1 in Groups 1 and 2, respectively (P = 0.02) (Figure 1a). The lymphocyte count measured at hospital admission showed significance in predicting poor maternal outcome, with an area under the curve (AUC) of 0.737 (95% CI: 0.578 to 0.897) and a cut-off value of 1,110 mcL (Figure 2a, Table 4). The sensitivity of this predictive value for poor maternal outcomes was 82%, and its specificity was 67%. Consistently, the mean maternal LDH at hospital admission was measured as 261.17 ± 56.1 U/L and 243.36 ± 111.8 U/L in the fetal complication group and noncomplication group, respectively (P < 0.05) (Figure 1b). ROC analysis showed LDH as a significant predictor of poor fetal outcome with a cut-off value of 213 U/L (AUC: 0.719; 95% CI: 0.566 to 0.872) (Figure 2b, Table 4). This value was calculated with 85% sensitivity and 60% specificity.

**Table 4:** AUCs of maternal lymphocyte and LDH AUC = area under the curve; LDH = lactate dehydrogenase. ^a^Under the nonparametric assumption. ^b^Null hypothesis: true area.

Test results variables	Area	Standard error^a^	Asymptotic significance^b^	Asymptotic 95% confidence interval	
				Lower limit	Upper Limit
Lymphocyte	0.737	0.081	0.016	0.578	0.897
LDH	0.719	0.078	0.012	0.566	0.872

## Discussion

In this retrospective study, we sought to determine any possible associations and predictive value of serum markers with maternal or fetal outcomes using ROC analyses. Our data revealed that the lymphocyte count was a significant predictor of poor maternal outcome and LDH was a significant predictor of poor fetal outcome.

Since the beginning of the COVID-19 pandemic, the lymphocyte count dynamics and inflammatory indices, including LDH, CRP, and IL-6, have been evaluated in several studies [13,14]. The slightly reduced lymphocyte count in nonpregnant adults was already identified as a biomarker to identify cases with a dismal prognosis requiring prompt intervention to improve outcomes [14]. In the present study, our findings showed that a decreased lymphocyte count within the recommended threshold of 1,110 mcL during a COVID-19-infected pregnancy was successful in predicting poor maternal prognosis. This value is important in identifying mothers who may deteriorate so that intervention is prompt. The most probable mechanisms are the lysis of lymphocytes during infection because of the expressed angiotensin-converting enzyme-2 (ACE 2) receptor on their surfaces, increased levels of cytokines, and the failure of lymphoid organs [14].

In a systematic review, the most seen abnormal biochemical parameters in pregnant women were increased CRP levels and decreased lymphocyte counts, similar to the age-matched nonpregnant group [15]. Our results showed that CRP was significantly higher in the maternal complication group. Benhamou et al. showed the possibility of thromboembolic complications because of elevated D-dimer concentrations in pregnancies infected with COVID-19 [16]. Similarly, in the present study, D-dimer levels in the maternal complication group were significantly higher than in the noncomplication group, putting them at greater risk for thromboembolism [17].

The prediction of poor fetal prognosis by LDH can be explained by chronic anoxia because of insufficient oxygenation in the placenta. LDH acts as an intracellular cytoplasmic enzyme in anaerobic glycolysis and is released into the circulation during cell death [18]. This enzyme also increases during preeclampsia, a well-known obstetric disease, because of potent glycolysis and chronic anoxemia as a result of poor placental perfusion [19]. Moreover, the LDH A gene is a well-defined hypoxia-related gene and correlates with hypoxia-inducible factor-1 alpha [19]. In the present study, our findings showed that increased LDH within the threshold of 213 U/L during a COVID-19-infected pregnancy was successful in predicting poor fetal prognosis. Therefore, maternal LDH may be the major candidate marker of the anoxia, cell damage, and placental dysfunction associated with the severity of COVID-19 infection. This enzyme may be of use in clinical practice to predict poor fetal prognosis.

In the present study, the rates of ICU requirement and maternal mortality were higher than reported previously in the literature. A systematic review of 192 studies including nearly 64,000 infected pregnant women demonstrated that ICU requirement and mortality rates were 3.3% and 0.8% among pregnant women, respectively, similar to age-matched nonpregnant women [4]. The reason for the higher rates in our study may be because of our tertiary hospital accepting moderate or severe COVID-19-infected pregnant women by referral; asymptomatic or mildly infected patients were not hospitalized.

Di Toro stated that the maternal course that would indicate hospitalization because of the COVID-19 infection usually occurs before delivery [20]. Although vaginal delivery is recommended, even with COVID-19 infection, cesarean section rates increased in our study, as in the literature [21]. In the present study, cesarean section was even performed on patients without maternal complications. Thus, we suspect that cesarean section was preferred in cases of maternal or fetal need and by health professionals to avoid transmission of COVID-19 although this is non-evidence-based.

In a systematic analysis study, preterm delivery was seen in 23% of patients with COVID-19 whereas the average rate ranges between 5% and 18% in noninfected patients according to the development of the countries [20,21]. In the present study, the gestational age during labor was 33.46 ± 3.8 weeks in the group with maternal complications, and this was significantly lower than the gestational age during labor without maternal complications. Maternal sepsis, the requirement for mechanical ventilation, and persistent respiratory distress may worsen fetal well-being and prompt the decision for emergency preterm delivery.

The mean birth weight was significantly lower in the maternal complication group. Although Poon et al. stated that pregnant patients with COVID-19 may have chronic hypoxia in earlier gestational weeks and recommended closer growth tracking, in the present study, all patients had deliveries during the acute phase of the infection [22]. We predict that the reason for the low birth weight in the group with maternal complications was because hypoxia in the acute period increases the rate of preterm birth rather than chronic hypoxia and intrauterine fetal growth retardation.

Based on the World Health Organization guidelines, it is preferable for the mother to breastfeed with a surgical mask from the first day because no virus has been clearly shown in breast milk [23]. Antibodies passing from breast milk may protect neonates from infection [23]. In the present study, we observed that breastfeeding began immediately only in one-quarter of the neonates. Similarly, Ferrazzi et al. reported that 28.6% of newborns were breastfed immediately, and two cases of mother-to-neonate transmission of COVID-19 were reported because of breastfeeding while not wearing a surgical mask [24]. In the present study, only one neonate who was delivered at term by cesarean section had vertical transmission. Because this newborn was fed with formula separate from the mother for the first five days, we consider its vertical transmission. In the literature, viral RNA was found in the peripheral blood specimen of infected nonpregnant women [25]. Thus, understanding the probability of mother-to-fetus transmission is possible given current knowledge. Di Toro et al. reported on 19 neonates with positive nasopharyngeal swabs in their meta-analysis [20]. Nevertheless, because these swabs were not taken immediately after delivery, the authors could not exclude the possibility of contagion during newborn follow-up. Notably, in case series data, Wu et al. demonstrated higher concentrations of immunoglobulin (Ig)-M and IgG in one newborn’s peripheral blood, which suggests intrauterine infection [26]. However, their case was COVID-19 PCR-negative, and the serum concentrations of IgM decreased immediately [26].

In the present study, we saw a high rate of admission to the neonatal ICU (80.49%). Previous studies have shown a low need for neonatal ICU, even lower rates than in term babies born to mothers without infection [27]. However, in the present study, preterm deliveries occurred at higher rates. Thus, neonatal ICU admissions seem to be for preterm-care purposes and requests to follow neonates separately from COVID-19-infected mothers. In addition, neonatal ICU admission indications may differ by clinic during this uncertain period.

In the present study, we observed three fetuses with intrauterine mortus (6.67%) and one abortion (2.22%) but no neonatal deaths. Fetal and neonatal mortality has been widely reported in the literature [20,28]. Mullins et al. analyzed 4,005 pregnant women who were suspected of having or being infected with COVID-19, and they found the overall rate of stillbirth to be 0.5% [29]. Similarly, Bellos et al. reviewed 16 observational studies and 44 case reports and saw low mortality rates [30]. Although our sample is small, the thromboembolic-enhancing effect of COVID-19 should be considered and makes us doubt that it causes stillbirth [16].

The strengths of our study are that the pregnant patients were from the same geographic area and a single center, which helped to standardize the management of the infection and antenatal and neonatal care. This study included only pregnant patients who were forced to deliver during acute infections of COVID-19. A limitation of the study is the difficulty of distinguishing whether clinical outcomes are because of the physiologic or pathologic changes of pregnancy or because of existing infection. Other limitations include the wider indications for cesarean section because of a non-evidence-based belief that vaginal delivery will increase respiratory distress and neonatal ICU admission, which shows physicians’ intent to protect neonates from the infection.

## Conclusions

To conclude, the study clearly demonstrates the association between elevated LDH, poor fetal prognosis, and decreased lymphocyte count with poor maternal prognosis in COVID-19-infected pregnancies. It is important to identify cases that may lead to complications or fatality within the first 24 hours of admission to the hospital. Evaluation of lymphocyte count and LDH combined with routine markers may aid early risk assessment, close monitoring, and rapid management of the infection.
